# Fournier's Gangrene as Seen in University of Maiduguri Teaching Hospital

**DOI:** 10.1155/2013/673121

**Published:** 2013-08-12

**Authors:** S. Aliyu, A. G. Ibrahim, N. Ali, A. M. Waziri

**Affiliations:** Department of Surgery, University of Maiduguri Teaching Hospital and College of Medical Sciences University of Maiduguri, PMB 1414, Maiduguri, Nigeria

## Abstract

*Background*. Fournier's gangrene is uncommon but increasingly being seen over the last two decades probably due to increasing socioeconomic problems including an upsurge in HIV infection especially in the tropics. *Patients and Methods*. The study retrospectively reviewed all patients with Fournier's gangrene managed in UMTH between January 2007 and December 2012. *Results*. Thirty-eight males aged 2 weeks to 80 years (mean 37.82) were reviewed, with most aged 30–39 years (13 (34.21%)). Clinical features were scrotal pain and swelling, 36 (94.74%), fever, 19 (50.00%), and discharging scrotal wound, 19 (50.00%). The predisposing conditions were UTI secondary to obstructive uropathy in 11 (28.95%), perianal suppuration, and HIV, in 8 (21.05%) patients each. Wound biopsy culture revealed mixed organisms in 27 (71.05%). Twenty-six (68.42%) had blood transfusions. Thirty-seven (97.37%) patients had wound debridement. Twenty (52.63%) had flap rotation for skin cover. There were 6 (15.79%) mortalities, of which 4 (10.53%) were HIV positive, 1 (2.63%) was diabetic, and 1 (2.63%) was both diabetic and HIV positive. *Conclusion*. Fournier's gangrene is a fulminant synergistic necrotising fasciitis of the perineum and genitalia with poor prognosis especially when associated with HIV and diabetes, requiringprompt and aggressive management for good outcome.

## 1. Introduction

Fournier's gangrene is a rare and often fulminant necrotising fasciitis of the perineum and genital region frequently due to a synergistic polymicrobial infection [1]. The condition was first described as a disease of young adults of unknown cause by Fournier in 1888 [2]. The disease process affects all ages and has been reported in both sexes [3]. Some systemic illnesses are associated with Fournier's gangrene. The most frequent of these are diabetes mellitus [4], HIV [5], and alcoholism [6]. Others are local conditions like perianal suppurations and urinary tract infection (UTI) complicating obstructive uropathy [7]. The study reviewed Fournier's gangrene in relation to aetiology, presentation, management, and outcome.

## 2. Patients and Methods

**Figure 1:**
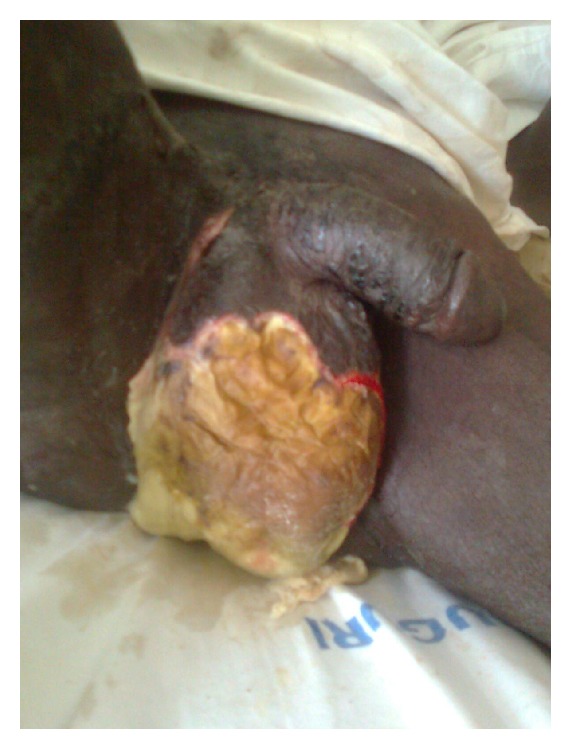
Fournier's gangrene predebridement.

**Figure 2:**
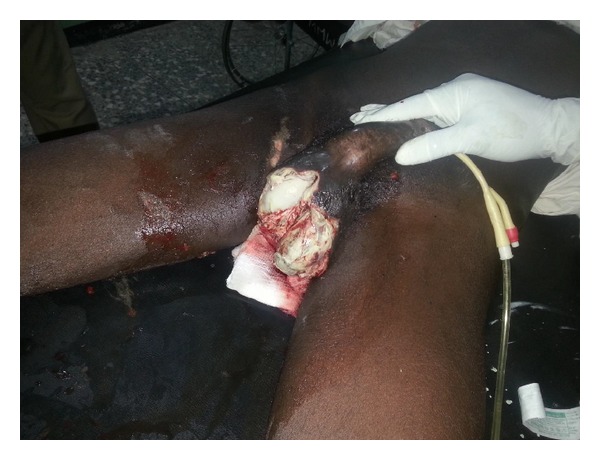
Fournier's gangrene postdebridement.

The study reviewed all patients managed for Fournier's gangrene in UMTH over a 6-year period between January 2007 and December 2012. A written informed consent was obtained from all patients, and written permission was given by the hospital Medical and Ethical Committee. Data were extracted from clinical and laboratory information and analysed using SPSS version 16. The diagnosis of Fournier's gangrene (Figures 1 and 2) was made on clinical assessment. Patients were resuscitated where necessary with antibiotics (metronidazole and ceftriaxone), intravenous fluids, analgesics, and antitetanus prophylaxis, blood transfusions were also given. Investigations carried out included packed cell volume, urinalysis, blood sugar and chemistry, wound swab and biopsy for microbial culture, and HIV screening. Wound debridement was done for all patients (some more than once) under general or spinal anaesthesia. The definitive management was done when the wound became clean with healthy granulation. The definitive management took the form of healing by secondary intention, by secondary closure, skin grafting, and skin flap rotation.

## 3. Results

A total of 43 patients were seen over the study period with five excluded on account of incomplete data, leaving 38 for detailed analysis. All patients were males aged 2 weeks to 80 years, with a mean of 37.82 years.  Table 1 shows the age distribution. The age group 30–39 years accounted for 13 (34.21%). Clinical features at presentation were scrotal pain and swelling in 36 (94.74%), fever in 19 (50.00%), scrotal wound discharge in 19 (50.00%), vomiting in 5 (13.16%), and dysuria in 4 (10.53%).  Table 2 showed predisposing conditions, with UTI secondary to obstructive uropathy (BPH/urethral stricture) in 11 (28.95%) patients, peri-anal suppuration, and HIV, in 8 (21.05%) each. Eleven (28.95%) patients were anaemic at presentation (PCV < 30%). Wound biopsy and culture revealed mixed organisms (aerobe and anaerobe) in 27 (71.05%), single isolates in 6 (15.79%), and sterile cultures in 5 (13.16%). Twenty-six (68.42%) had blood transfusions, 1 or 2 units in 16 (42.11%) patients, while 10 (26.32%) patients had 3 or 4 units transfused. Twenty-eight patients (73.68%) had wound debridement once or twice while 9 (23.68%) patients had 3 or more serial debridements. Twenty (52.63%) had flap rotation for skin cover, 4 (10.53%) had skin graft, while in 12 (31.58%) patients skin was covered by secondary closure, and 2 (5.26%) were allowed to heal by secondary intention. The average hospital stay was 4 weeks with a range of 1 to 7 weeks. There were 6 (15.79%) mortalities of which 4 (10.53%) were HIV positive, 1 (2.63%) was diabetic, while 1 (2.63%) was both diabetic and HIV positive. The causes of death were septicaemia and shock in 4 (10.53%), diabetic ketoacidosis, and acute renal failure in 1 (2.63%) each.

## 4. Discussion

**Figure 3:**
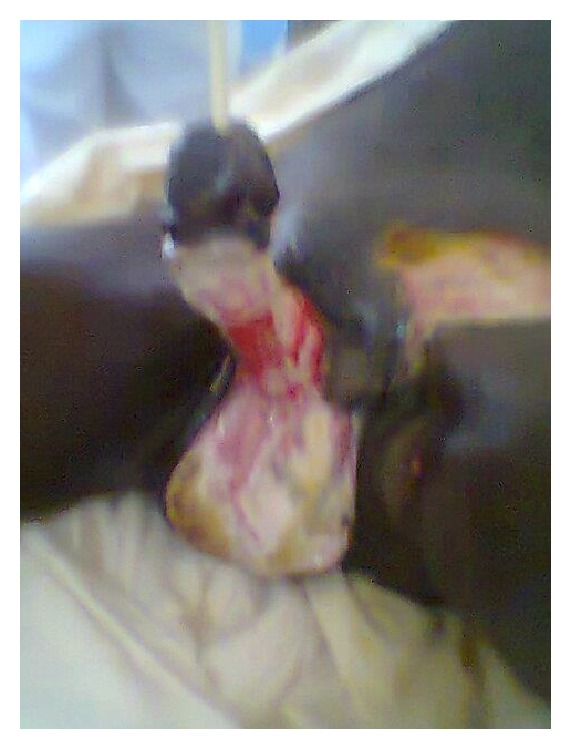
More Florid Fournier's in HIV.

Fournier's gangrene is a rare and often fulminant necrotising fasciitis of the perineum and genital region frequently due to a synergistic polymicrobial infection. It affects both sexes and all ages [5]. In this study all patients are males with peak age group 30–39 years. Clinical features of scrotal pain and swelling, fever, and scrotal discharge and sloughing were in keeping with similar studies [8]. Systemic diseases associated with Fournier's gangrene were diabetes, HIV, and alcoholism; however, this study did not find alcoholism to be associated with Fournier's. Local conditions like peri-anal suppuration and UTI [7] were found to be associated with Fournier's gangrene. Majority of the wound biopsy culture revealed polymicrobial infection in keeping with polymicrobial synergy. The management of this condition requires urgent and aggressive resuscitative methods with broad spectrum antibiotics, which covers both aerobes and anaerobes, as well as fluids, which may include blood transfusion if necessary [9]. The initial management for all patients in this study was similar. All patients had serial wound debridement in keeping with the standard in the management of this condition [7]. Some authors advocate suprapubic cystostomy and diverting colostomy to prevent wound contamination [10], and in this we had no cause to undertake such procedures. Wound closure is advocated once the wound is clean and this reduced hospital stay [11]. In this study the majority of the patients had wound closure (in keeping with the previous principle), by secondary closure, skin grafting, and flap rotation. The prognosis is good especially in children and young adults [12–14]; however, when it is associated with systemic diseases such as HIV (Figure 3) and uncontrolled diabetes, the prognosis is dismal as evidenced by high mortality rate in this study among HIV and diabetic patients, as opposed to findings by other authors that diabetes [15–18] has no adverse effect on survival in the disease.

## Figures and Tables

**Table 1:** Age distribution.

Age	Frequency (%)
<20	2 (5.26)
20–29	4 (10.53)
30–39	13 (34.21)
40–49	9 (23.68)
50–59	4 (10.53)
60–69	5 (14.16)
≥70	1 (2.63)

Total	38 (100)

**Table 2:** Predisposing conditions.

Predisposing conditions	Frequency (%)
UTI in BPH/urethral stricture	11 (28.95)
Perianal suppurations	8 (21.05)
Abscess (4)	
Fistula (2)	
Fissure (1)	
Thrombosed haemorrhoids (1)	
HIV	8 (21.05)
Diabetes	6 (15.79)
SCD	1 (2.63)
Dermatitis	1 (2.63)
Omphalitis	1 (2.63)
